# Erratum to: Translational Modeling in Schizophrenia: Predicting Human Dopamine D_2_ Receptor Occupancy

**DOI:** 10.1007/s11095-016-1888-2

**Published:** 2016-03-10

**Authors:** Martin Johnson, Magdalena Kozielska, Venkatesh Pilla Reddy, An Vermeulen, Hugh A. Barton, Sarah Grimwood, Rik de Greef, Geny M. M. Groothuis, Meindert Danhof, Johannes H. Proost

**Affiliations:** Department of Pharmacokinetics, Toxicology and Targeting, Groningen Research Institute of Pharmacy, University of Groningen, Antonius Deusinglaan 1, 9713 AV Groningen, The Netherlands; Clinical Pharmacology & Pharmacometrics, Janssen Research and Development, A Division of Janssen Pharmaceutica NV, Beerse, Belgium; Worldwide Research & Development, Pfizer, Inc., Groton, Connecticut USA; Quantitative Solutions, Pivot Park, Molenweg 79, 5349 AC Oss, The Netherlands; Division of Pharmacology, Leiden Academic Center for Drug Research, Leiden, The Netherlands; AstraZeneca, Cambridge, UK; Institute of Engineering, Hanze University of Applied Sciences, Assen, The Netherlands

**Erratum to: Pharm Res**

**DOI 10.1007/s11095-015-1846-4**

There occurred two errors in the above manuscript (Pharm Res. 2015 Dec 30 [Epub ahead of print]). The correct information is as follows:

1. The differential equations in Appendix 2 should read:
  $$ \begin{array}{l}\begin{array}{l}\mathrm{d}\left({\mathrm{A}}_{\mathrm{bv}}\right)/\mathrm{d}\mathrm{t}=\left({\mathrm{CL}}_{\mathrm{bv}}/{\mathrm{V}}_{\mathrm{plasma}}\right)\ast {\mathrm{A}}_{\mathrm{plasma}}-\left({\mathrm{CL}}_{\mathrm{bv}}/{\mathrm{V}}_{\mathrm{bv}}\right)\ast {\mathrm{A}}_{\mathrm{bv}}-\left({\mathrm{CL}}_{\mathrm{bev}}/{\mathrm{V}}_{\mathrm{bv}}\right)\ast {\mathrm{fu}}_{\mathrm{plasma}}\ast {\mathrm{A}}_{\mathrm{bv}}\hfill \\ {}+\left({\mathrm{CL}}_{\mathrm{bev}}/{\mathrm{V}}_{\mathrm{bev}}\right)\ast {\mathrm{fu}}_{\mathrm{brain}}\ast {\mathrm{A}}_{\mathrm{bev}}+\left({\mathrm{CL}}_{\mathrm{eff}}/{\mathrm{V}}_{\mathrm{bev}}\right)\ast {\mathrm{fu}}_{\mathrm{brain}}\ast {\mathrm{A}}_{\mathrm{bev}}\hfill \end{array}\hfill \\ {}\begin{array}{l}\mathrm{d}\left({\mathrm{A}}_{\mathrm{bev}}\right)/\mathrm{d}\mathrm{t}=\left({\mathrm{CL}}_{\mathrm{bev}}/{\mathrm{V}}_{\mathrm{bv}}\right)\ast {\mathrm{fu}}_{\mathrm{plasma}}\ast {\mathrm{A}}_{\mathrm{bv}}-\left({\mathrm{CL}}_{\mathrm{bev}}/{\mathrm{V}}_{\mathrm{bev}}\right)\ast {\mathrm{fu}}_{\mathrm{brain}}\ast {\mathrm{A}}_{\mathrm{bev}}-\left({\mathrm{CL}}_{\mathrm{eff}}/{\mathrm{V}}_{\mathrm{bev}}\right)\ast {\mathrm{fu}}_{\mathrm{brain}}\ast {\mathrm{A}}_{\mathrm{bev}}\hfill \\ {}-\left({\mathrm{CL}}_{\mathrm{st}}/{\mathrm{V}}_{\mathrm{bev}}\right)\ast {\mathrm{fu}}_{\mathrm{brain}}\ast {\mathrm{A}}_{\mathrm{bev}}+\left({\mathrm{CL}}_{\mathrm{st}}/{\mathrm{V}}_{\mathrm{st}\mathrm{f}}\right)\ast {\mathrm{fu}}_{\mathrm{brain}}\ast {\mathrm{A}}_{\mathrm{st}\mathrm{f}}\hfill \end{array}\hfill \\ {}\begin{array}{l}\mathrm{d}\left({\mathrm{A}}_{\mathrm{st}\mathrm{f}}\right)/\mathrm{d}\mathrm{t}=\left({\mathrm{CL}}_{\mathrm{st}}/{\mathrm{V}}_{\mathrm{bev}}\right)\ast {\mathrm{fu}}_{\mathrm{brain}}\ast {\mathrm{A}}_{\mathrm{bev}}-\left({\mathrm{CL}}_{\mathrm{st}}/{\mathrm{V}}_{\mathrm{st}\mathrm{f}}\right)\ast {\mathrm{fu}}_{\mathrm{brain}}\ast {\mathrm{A}}_{\mathrm{st}\mathrm{f}}-\mathrm{k}\mathrm{o}\mathrm{n}\ast {\mathrm{fu}}_{\mathrm{brain}}\ast {\mathrm{A}}_{\mathrm{st}\mathrm{f}}\ast \left({\mathrm{B}}_{\max }-\mathrm{C}\mathrm{B}\right)\hfill \\ {}+\mathrm{koff}\ast {\mathrm{A}}_{\mathrm{st}\mathrm{b}}\hfill \end{array}\hfill \\ {}\mathrm{d}\left({\mathrm{A}}_{\mathrm{st}\mathrm{b}}\right)/\mathrm{d}\mathrm{t}=\mathrm{k}\mathrm{o}\mathrm{n}\ast {\mathrm{fu}}_{\mathrm{brain}}\ast {\mathrm{A}}_{\mathrm{st}\mathrm{f}}\ast \left({\mathrm{B}}_{\max }-\mathrm{C}\mathrm{B}\right)-\mathrm{ko}\mathrm{f}\mathrm{f}\ast {\mathrm{A}}_{\mathrm{st}\mathrm{b}}\hfill \end{array} $$
2. Table III should read:

**Table III:** *In vitro*, *in vivo* and *ex vivo* values estimates used in the human D_2_RO predictive model.

	Clozapine	Haloperidol	Olanzapine	Paliperidone	Quetiapine	Risperidone
Fraction unbound in brain	0.011 ^a^	0.023 ^b^	0.034 ^a^	0.0755 ^c^	0.025 ^a^	0.0699 ^c^
Fraction unbound in plasma	0.0300 ^d^	0.0800 ^e^	0.0700 ^m^	0.226 ^f^	0.170 ^e^	0.100 ^f^
Approach A: Human D_2_RO predictions based on *in vitro* information						
Papp × 10^−6^ (cm/s)	28.3 ^a^	28.6 ^a^	15.7 ^a^	16.8 ^g^	33.0 ^a^	19.8 ^g^
CL_bev_ (L/h) derived from Papp	20.4	20.6	11.3	7.80	23.8	12.96
Efflux ratio	–	–	–	2.10 ^g^	–	1.20 ^g^
CL_eff_ (L/h) based on ER	–	–	–	8.58	–	2.59
*In vitro* Ki (nM)	82.0 ^h^	0.700 ^h^	5.10 ^i^	2.075 ^e^	155 ^h^	2.175 ^e^
k_off_ (h^−1^)	83.16 ^h^	1.02 ^h^	2.34 ^h^	1.56 ^j,n^	180.78 ^h^	1.56 ^j^
Approach B: Human D_2_RO predictions based on *in vivo* information						
Papp_calc_ × 10^−6^ (cm/s)	–	–	100	493 ^n^	–	493
CL_bev_ (L/h) based on Papp_calc_	–	–	72.2	355 ^n^	–	355
CL_eff_ (L/h) scaled from rat	–	–	NA	11594	–	2486
k_off_ (h^−1^)	–	–	3.04 ^k^	0.671 ^l,n^	–	0.671 ^l^
Approach C: Human D_2_RO predictions integrating *in vitro* and *in vivo* information						
Corrected *In vivo* Kd (nM)	–	–	4.38	0.352	–	0.395

*NA* Not applicable

^a^Reference (32); ^b^ Reference (33); ^c^ Reference (34); ^d^ Reference (35); ^e^ In-house values; ^f^ Reference (36); ^g^ Reference (37);

^h^Reference (38); ^i^ Reference (39); ^j^ Reference (5); ^k^ Reference (7); ^l^ Reference (8); ^m^ Reference (40); ^n^Assumed to be equal to risperidone

